# Mechanical Behavior and Failure Mode of Steel–Concrete Connection Joints in a Hybrid Truss Bridge: Experimental Investigation

**DOI:** 10.3390/ma13112549

**Published:** 2020-06-03

**Authors:** Yingliang Tan, Bing Zhu, Le Qi, Tingyi Yan, Tong Wan, Wenwei Yang

**Affiliations:** 1 Department of Bridge Engineering, School of Civil Engineering, Southwest Jiaotong University, Chengdu 610031, China; yingliang_tan@my.swjtu.edu.cn (Y.T.); qileswjtu@126.com (L.Q.); tingyi_yan@yeah.net (T.Y.); wantongswjtu@163.com (T.W.); 2 Department of Civil Engineering, School of Civil and Hydraulic Engineering, Ningxia University, Yinchuan 750021, China; nxyangww@126.com

**Keywords:** hybrid truss bridge, steel–concrete connection joint, mechanical behavior, failure mode, strain, static test

## Abstract

The core part of a hybrid truss bridge is the connection joint which combines the concrete chord and steel truss-web members. To study the mechanical behavior and failure mode of steel–concrete connection joints in a hybrid truss bridge, static model tests were carried out on two connection joints with the scale of 1:3 under the horizontal load which was provided by a loading jack mounted on the vertical reaction wall. The specimen design, experimental setup and testing procedure were introduced. In the experiment, the displacement, strain level, concrete crack and experimental phenomena were factually recorded. Compared with the previous study results, the experimental results in this study demonstrated that the connection joints had the excellent bearing capacity and deformability. The minimum ultimate load and displacement of the two connection joints were 5200 kN and 59.01 mm, respectively. Moreover, the connection joints exhibited multiple failure modes, including the fracture of gusset plates, the slippage of high-strength bolts, the local buckling of compressive splice plates, the fracture of tensile splice plates and concrete cracking. Additionally, the strain distribution of the steel–concrete connection joints followed certain rules. It is expected that the findings from this paper may provide a reference for the design and construction of steel–concrete connection joints in hybrid truss bridges.

## 1. Introduction

Steel–concrete composite bridges are widely used because they combine the advantages of prestressed concrete box girder bridges and steel truss bridges. A composite bridge with steel truss members instead of traditional concrete webs attracted the attention of researchers, which was also known as a hybrid truss bridge (HTB). HTBs have a lighter weight and smaller beam height than prestressed concrete box girder bridges, enhancing the bridge span. 

The Arbois Bridge [1], which was built in France in 1985, was the first attempt of this type of bridge in the world. Additionally, more representative HTBs have been built in France, such as Boulonnais Viaducts [2] and Bras de la Plaine Bridge [3]. Many hybrid truss bridges have been designed and built in other countries in Europe, including Lully Viaduct [4], Dreirosen Bridge [5], Europe Bridge [6], and Nantenbach Railroad Bridge [7,8]. In recent years, some countries in Asia have also begun to study this kind of bridge and put it into practice [9,10,11,12,13,14,15]. However, the study of HTBs in China started late, and the first HTB, Jiangshan Bridge, was built in 2012 [16]. Although the same type of structure was applied to several bridges (e.g., Minpu Bridge [17], Houhecun Bridge [18], and Deshenglu Bridge [19]), the research on HTB was not comprehensive.

The steel–concrete connection joint is considered the most important part of hybrid truss bridges, transferring the load between the concrete chord and steel truss web [11,17,20,21,22,23,24]. Therefore, some experimental and analytical studies on such joint structures have been reported. At the end of the 20th century and the beginning of the 21st century, Japanese scholars conducted various types of research to investigate the mechanical properties of connection joints [12,15,25,26,27,28]. Additionally, the stress transfer mechanism and mechanical characteristic of two types of steel–concrete connection joints were compared by Sato et al. [29]. The results showed that the connection joint with the perfobond rib (PBL) shear connectors had a greater bearing capacity than that of the joint with welded headed studs. In the early studies, the welding process was widely recommended and applied in the joint structures. However, the mechanical behavior was affected by the form of welding [29]. To reduce welding during construction, Jung et al. [1,11,13,20,22] proposed a new connection joint composed of connection plates and a connection bolt. Furthermore, experimental and numerical investigations were carried out on this new connection system and HTB girders to clarify the structural capacity, fatigue capacity and torsional behavior. Additionally, Zhou et al [18], Yin et al. [23,30] and Tan et al. [24] introduced another joint with high-strength bolts to decrease welding and conducted static model tests to investigate the connection performance of such joints. Their results clarified that the connection joint with high-strength bolts had excellent bearing capacities and safety reserves. However, the failure mode of the steel–concrete connection joint remains controversial. Zhou et al [18] reported that such joints failed because of the local buckling and fracture of gusset plates, while Yin et al. [23,30] found that the local buckling of steel truss-web members was one of the main failure modes. Moreover, what is less clear is the mechanical behavior of such joints at each loading step. In particular, the strain distribution rule of the main components is unclear. Hence, it is necessary to carry out the model test to investigate the mechanical behavior and failure mode of connection joints in detail.

In this paper, we sought to investigate the mechanical behavior and failure mode of steel–concrete connection joints. More specifically, this study aimed to determine the following specific research directions: (1) the ultimate bearing capacity and corresponding displacement of the proposed joints, (2) the typical failure mode of such joints, (3) the strain distribution rule of the main components, and (4) the key component of the steel–concrete connection joint to carry the external load. Therefore, we conducted static loading tests on two joint specimens with the scale of 1:3. Such experimental investigations of the proposed joint form may enrich the experimental data and provide an experimental reference for the design and construction of such joints in hybrid truss bridges.

## 2. Experimental Program

### 2.1. Specimens

**Figure 1:**
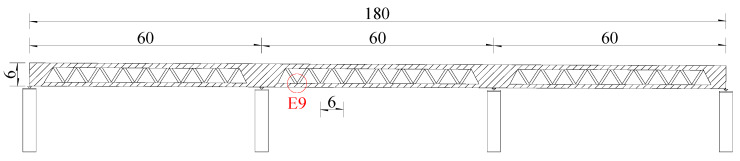
The most unfavorable joint (unit: m). E9: the ninth joint of the lower chord from left to right.

As shown in Figure 1, two specimens with the scale of 1:3 were designed on the basis of the typical steel–concrete connection joint E9, which was selected from the preliminary design of the first hybrid continuous truss bridge in Chinese railway bridges.

**Figure 2:**
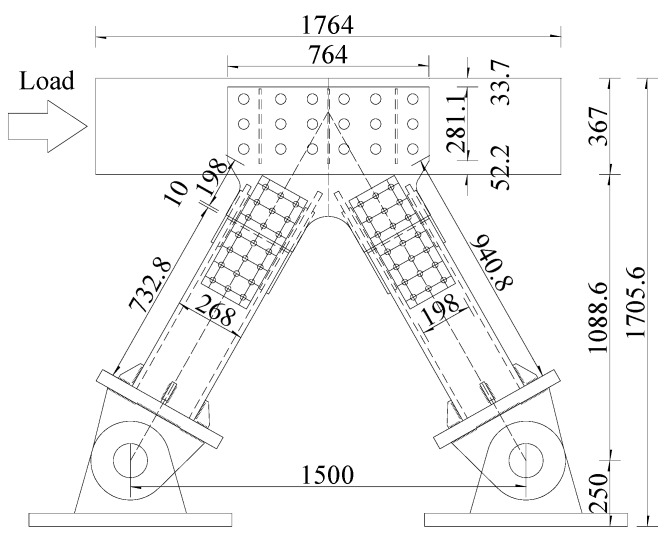
Schematic diagram of the specimen (unit: mm).

Figure 2 shows the schematic diagram of the joint specimens. Furthermore, Specimen 1 (Southwest Jiaotong University, Chengdu, China) and Specimen 2 (Southwest Jiaotong University, Chengdu, China) shared the same model parameters. The steel–concrete joint consists of the concrete chord (1764 mm × 334 mm× 367 mm), gusset plates ((CRTB) Tycoon Industrial Development Co., Ltd., Baoji, China) (764 mm × 629 mm× 16 mm) perforated 18 holes (40 mm in diameter), PBL shear connectors (Southwest Jiaotong University, Chengdu, China) (12 mm in diameter), steel truss-web members (268 mm× 184 mm× 20 mm), steel reinforcements (12 mm in diameter), and rectangular stirrups (8 mm in diameter). The material properties of C50 concrete (Sichuan Southwest Cement Co., Ltd., Chengdu, China), Q370qE steel (Baoshan Iron & Steel Co., Ltd., Shanghai, China), and HRB400 steel (Baoshan Iron & Steel Co., Ltd., Shanghai, China) are listed in Table 1.

### 2.2. Experimental Setup and Testing Procedure

**Figure 3:**
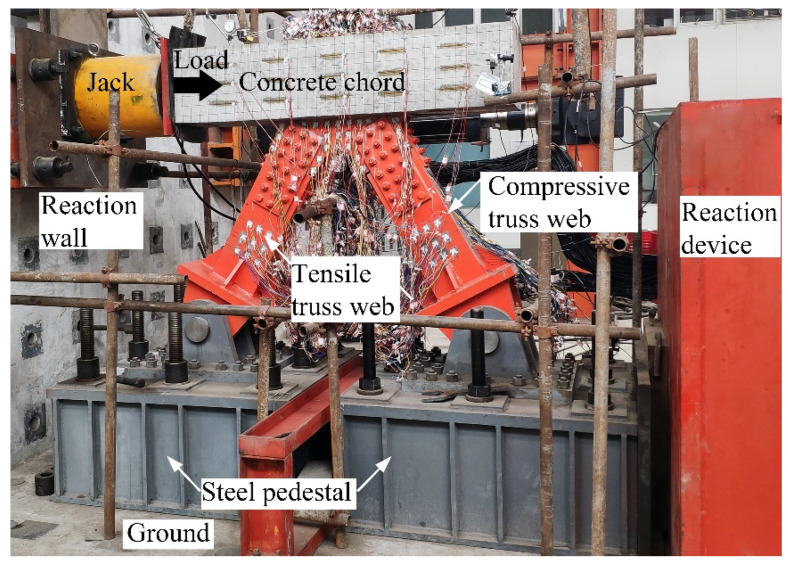
Experimental setup.

The static tests of the steel–concrete connection joints were carried out in the National Engineering Laboratory for Technology of Geological Disaster Prevention in Land Transportation, and the experimental setup is shown in Figure 3. To provide enough reaction force, two steel pedestals and a reaction device were installed on the ground. The steel pedestals with the hinge supported the connection joints. The horizontal load was offered by the loading jack with a capacity of 6300 kN, acting on one end of the concrete chord, whose direction was shown by the solid arrow in the Figure 3.

**Figure 4:**
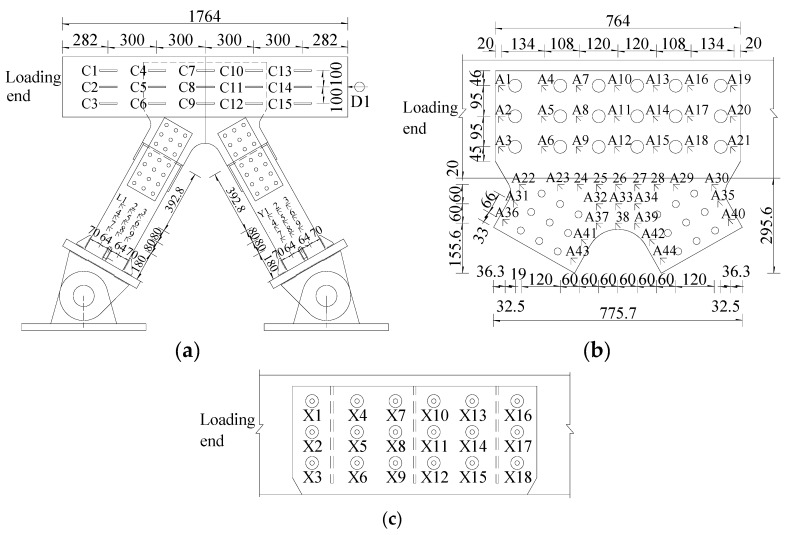
Arrangement of the displacement meters and strain gauges (unit: mm). (**a**) The concrete chord and steel truss-web members; (**b**) the gusset plates; and (**c**) the PBL shear connectors. C1: No. 1 strain gauge of concrete chord; A1: No. 1 strain gauge of gusset plates; X1: No. 1 strain gauge of PBL shear connectors.

Before the formal multi-step loading, pre-loading was performed to avoid assembly clearance. The loading step of 400 kN was adopted within 0–2000 kN. Then, when the horizontal load was in the range of 2000–3000 kN, the loading step was reduced to 200 kN. Finally, the loading step of 100 kN was applied until the end of the loading procedure. The laser displacement sensor (D1) was used for measuring the horizontal displacement in the loading direction. Furthermore, the strain gauges were installed to monitor the strain of the main components, including the concrete chord, gusset plates, PBL shear connectors, and the steel truss-web members, as shown in Figure 4. 

## 3. Experimental Results and Discussions

### 3.1. Experimental Phenomena and Failure Modes

**Figure 5:**
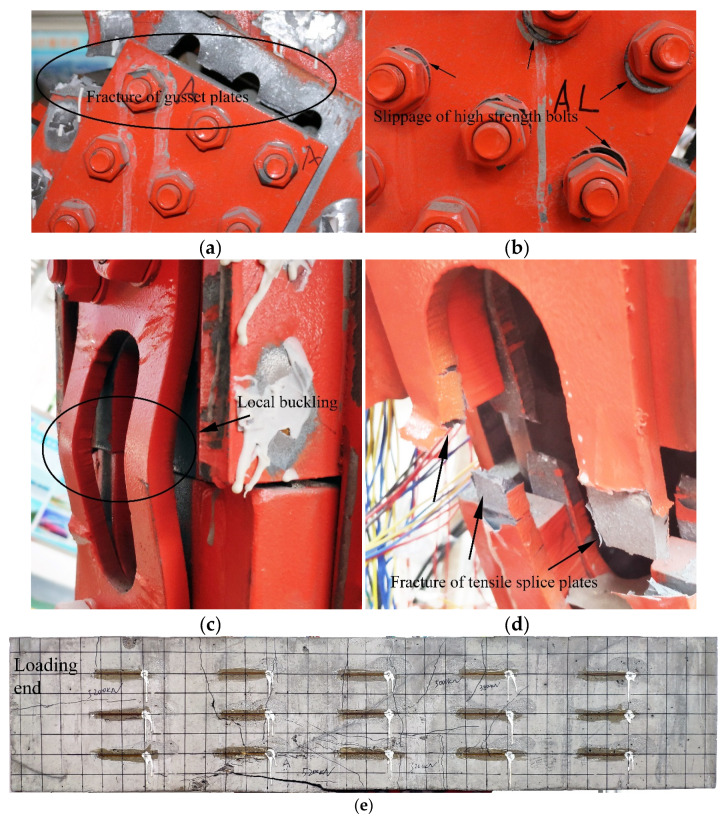
Failure modes of Specimen 1: (**a**) the fracture of gusset plates; (**b**) the slippage of high-strength bolts; (**c**) the local buckling of the compressive splice plates; (**d**) the fracture of the tensile splice plates; and (**e**) the concrete cracking.

**Figure 6:**
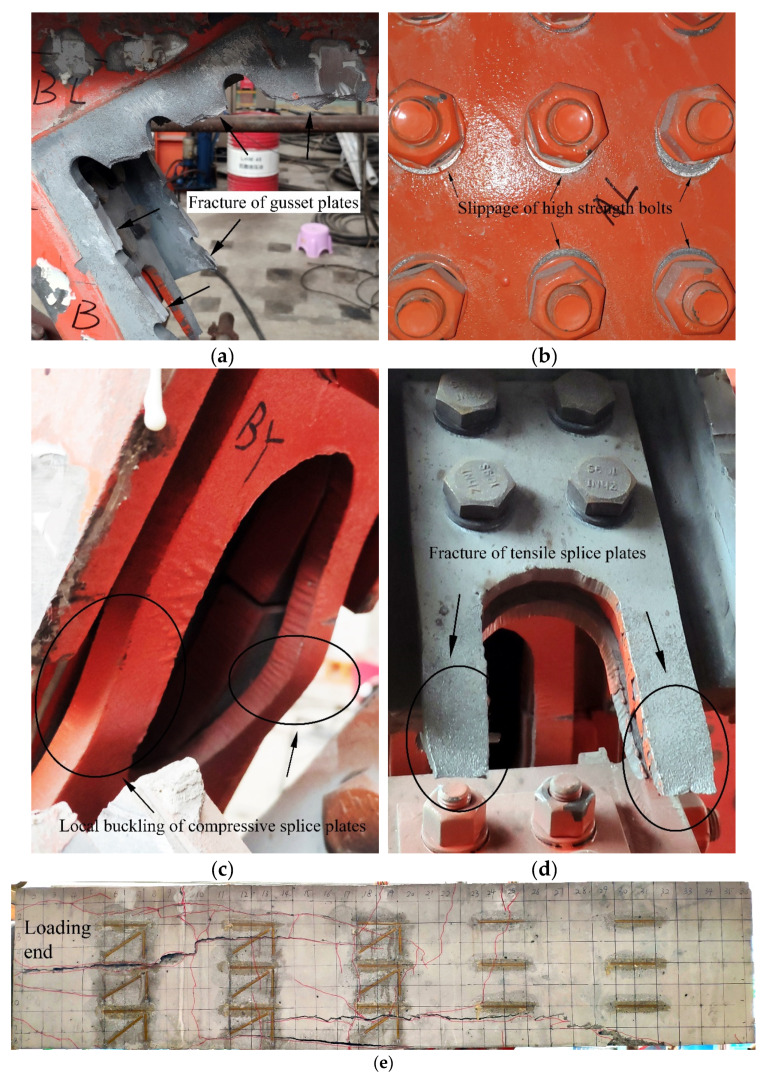
Failure modes of Specimen 2: (**a**) the fracture of the gusset plates; (**b**) the slippage of the high-strength bolts; (**c**) the local buckling of the compressive splice plates; (**d**) the fracture of the tensile splice plates; (**e**) the concrete cracking.

To precisely investigate the failure modes of the proposed connection joints, the experimental phenomena and data were recorded in detail. Figure 5 and Figure 6 present the failure modes of Specimen 1 and Specimen 2, respectively. For Specimen 1, there was no macroscopic damage until the applied load reached 3800 kN. Then, the relative location between the gusset plates and the splice plates was changed on the tension side. Additionally, the initial crack with the length of 170 mm appeared on the concrete chord and which did not develop any more in the subsequent loading procedure. At the load of 4400 kN, local buckling of the splice plate on the compressive side was observed, as shown in Figure 5c. Then, in Figure 5b, the visible slips of high-strength bolts were noticed at the load of 4800 kN, which indicated that the high-strength bolts were out of action. When the applied load reached 5200 kN, a lot of cracks appeared on the concrete chord, as shown in Figure 5e. Finally, two noises were heard within seven seconds due to the fracture of the gusset plates and splice plates with hand holes (in Figure 5a,d), meanwhile, Specimen 1 lost its bearing capacity to resist the external load.

For Specimen 2, the first crack of the concrete chord emerged near the loading end at the load of 3400 kN, but this crack did not spread in the latter loading stages. Furthermore, the relative location malposition between the gusset plates and the splice plates with hand holes was also observed. Then, the local buckling of the compressive splice plates and the slippage of high-strength bolts were observed, as shown in Figure 6c,b. At the peak load of 5400 kN, it can be seen from Figure 6e that some cracks severely developed. Finally, the gusset plates suddenly broke completely causing a sound to be heard and Specimen 2 lost its bearing capacity, as shown in Figure 6a. Meanwhile, looking at Figure 6d, the splice plates with hand holes were also pulled off.

Based on the experimental observation of two specimens, the order of the destruction course of the proposed connection joints was the local buckling of the compressive splice plates, the slippage of high-strength bolts, the cracking of the concrete chord, the fracture of the gusset plates and the tensile splice plates. Clearly, two specimens showed similar test phenomena and failure modes. Nonetheless, the damage degree of Specimen 2 was greater than that of Specimen 1, for example, the gusset plates of Specimen 2 were pulled to the point of total fracture. The difference in the experimental results between the two specimens is attributable to the fact that the first fracture of Specimen 1 dissipated part of the energy, making Specimen 1 unable to continue to bear a greater load, and also causing the damage degree to be less than that of Specimen 2. 

Compared with previous tests [11,18,23,30,31,32], one interesting finding from Table 2 was that the fracture of gusset plates and the slippage of high-strength bolts seemed to be two particular failure modes of such connection joints with high-strength bolts. A possible explanation for this might be that the cross-sectional area of the gusset plate was reduced due to the bolt holes, resulting in stress concentration. In addition, the two specimens in this study showed other failure modes, including the local buckling of compressive splice plates, the fracture of tensile splice plates, and concrete cracking. In particular, in contrast to previous studies, there were wide and long cracks on the concrete chord, which indicated that the performance of the concrete chord was also fully exerted in this study. To make the assembly process easy, it was necessary to drill hand holes on some splice plates, which caused these splice plates to buckle or fracture. This is difficult to avoid in the type of scale model unless these splice plates are not set here. Moreover, as Table 2 shows, for the steel–concrete connection joints with the high-strength bolts, the failure modes of such joints are related to the relative thickness of the gusset plates and truss-web members. For example, for specimens RGP (Rectangular gusset plate is used in the specimen), the increase in the thickness of steel truss-web members caused the failure mode to change from the failure of steel truss web to the failure of gusset plates and high-strength bolts. On the other hand, for the embedded type joints (joint type B), if the steel web members are not damaged, the specimens will fail due to the cracking of the concrete chord.

### 3.2. Load–Displacement Curves

**Figure 7:**
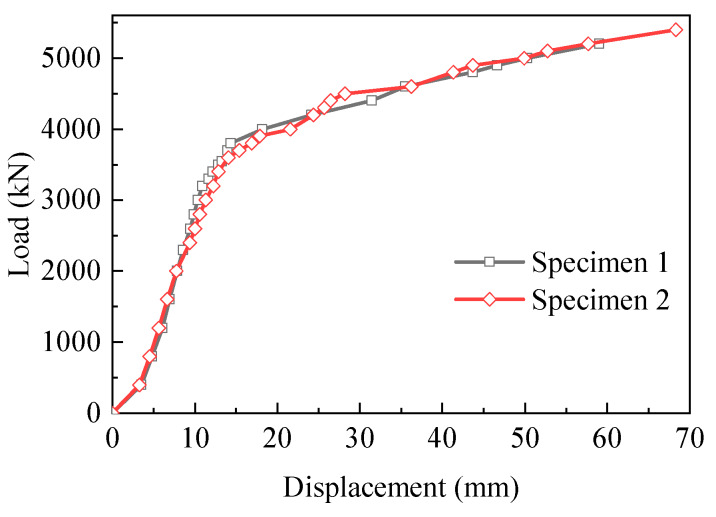
Load–displacement curves of the two specimens.

Figure 7 presents the relation between the applied load and the horizontal displacement. As shown in Figure 7, two load–displacement curves were almost coincident. The corresponding displacement linearly increased with the increase in the applied load in the initial stage. Specimen 1 began to yield, and the curve started to flatten at the load of 3200 kN. With the increase in the load, the exposed gusset plates were pulled to fracture and the composite joint failed to carry the applied load. Hence, the peak load of 5200 kN was regarded as the bearing capacity of Specimen 1. In contrast to Specimen 1, the ultimate capacity of Specimen 2 reached 5400 kN.

Table 3 provides a comparison of the characteristic loads and displacements between this study and previous studies [18,23]. RGP specimens and SJ specimens had a similar design to the specimens in this test, and they were all scale modes with a scale ration of 1:3. The minimum yield load and ultimate load of the specimens in this study were 3200 kN and 5200 kN, respectively. No significant difference in the yield load was found between the RGP specimens and the specimens in this study. However, the ultimate bearing capacity of the composite joints in this test was significantly greater than that of the RGP specimens and SJ specimens. Additionally, the minimum value of the ultimate displacement was 59.01 mm. The comparison of the results in Table 3 indicated that Specimen 1 and Specimen 2 had the greatest bearing capacity and deformability. For example, without considering the steel grade, the ultimate bearing capacity and displacement of Specimen 1 increased by 16% and 36%, respectively, compared with those of Specimen RGP-3, due to the increase in the thickness of the gusset plate from 12 to 16 mm.

### 3.3. Load–Strain Curves of the Concrete Chord

**Figure 8:**
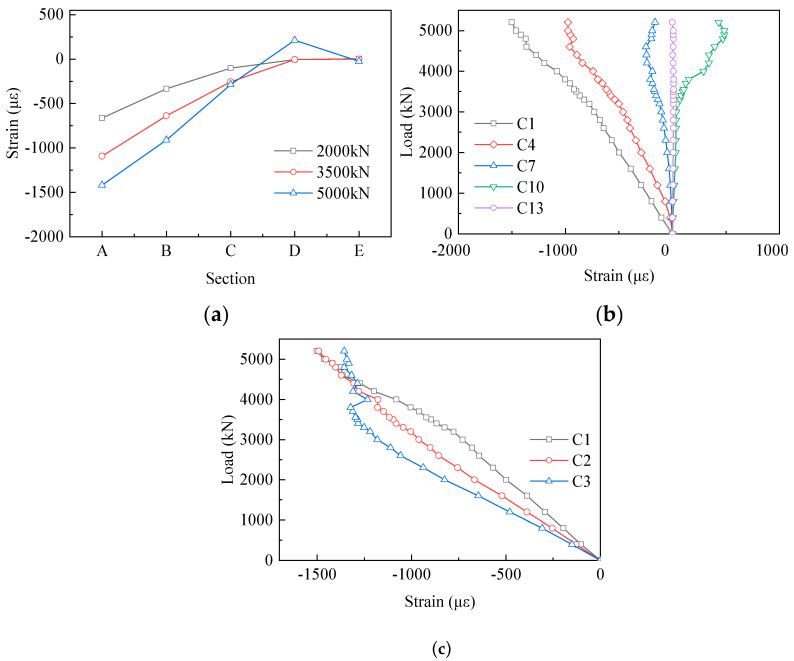
Strain results of the concrete chord: (**a**) the average strain values of each section; (**b**) the first row of the strain gauges; (**c**) the first column of the strain gauges.

Specimen 1 was selected as the narrative object to avoid redundancy in the following sections. The strain results of the concrete chord are set out in Figure 8. To minimize the influence of the strain gauge failure, the average strain values of each section in the elastic state (2000 kN), elastic-plastic state (3500 kN), and closely ultimate state (5000 kN) are shown in Figure 8a. There is a clear trend of reducing in the compressive strain values. Evidently, concrete near Section A suffered from the biggish axial force. At the load of 2000 kN, the compressive strains of Section B, C and D were 50%, 15% and 1% of the strain of Section A (−663.14 µε). The average strain values decreased quickly along the loading direction, because PBL shear connectors transferred force efficaciously from the concrete chord to the gusset plate. Moreover, the area of the concrete chord close to Section E was almost not subjected to the load. Figure 8b,c presents the strain values of the first row and the first column of gauges, respectively. There was a rapid decrease in the compressive strain values along the loading direction on the concrete chord. For example, the strain values of C1, C4, C7, C10 and C13 were −498.32 µε, −291.49 µε, −49.06 µε, 29.82 µε, and 6.73 µε, respectively, at a load level of 2000 kN. Conversely, in the vertical direction, the strain values of C2 and C3 were 1.34 times and 1.66 times the strain of C1 (−498.32 µε). There was a significant increase in the strains from the top to the bottom of the concrete chord.

### 3.4. Load–Strain Curves of the Gusset Plates

**Figure 9:**
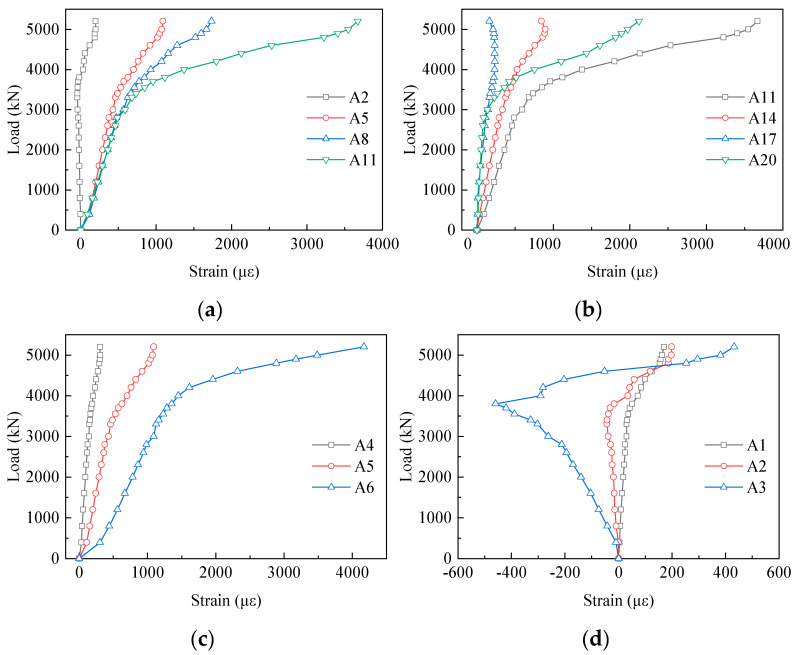
Load–strain curves of the embedded gusset plates: (**a**) A2 to A11 in the second row; (**b**) A11 to A20 in the second row; (**c**) the second column of strain gauges; and (**d**) the first column of the strain gauges.

Figure 9 shows the load–strain curves of the gusset plates which were wrapped by the concrete chord. In the second row, the strain values of A2, A5, A8, A14, and A17 were 6%, 30%, 47%, 25%, and 6% of that of A11 at approximately 5000 kN, respectively. What stands out in Figure 9a,b is the rapid increase in strains from A2 to A11 and the significant decrease in that from A11 to A20. In other words, the strain results of the embedded gusset plates presented a trend of increasing first and then decreasing along the loading direction. Furthermore, almost all the points did not reach the yield strain (2194 µε), except for A11. In the vertical direction, in Figure 9c there was an evident tendency of increasing from A4 to A6 (taking the second column strain gauges as an example). Moreover, the same phenomena occurred in other columns. Exceptionally, in Figure 9d compressive strains occurred at the first column of measuring points, indicating that the front ends of the gusset plates were biased towards compression at the early loading stage.

**Figure 10:**
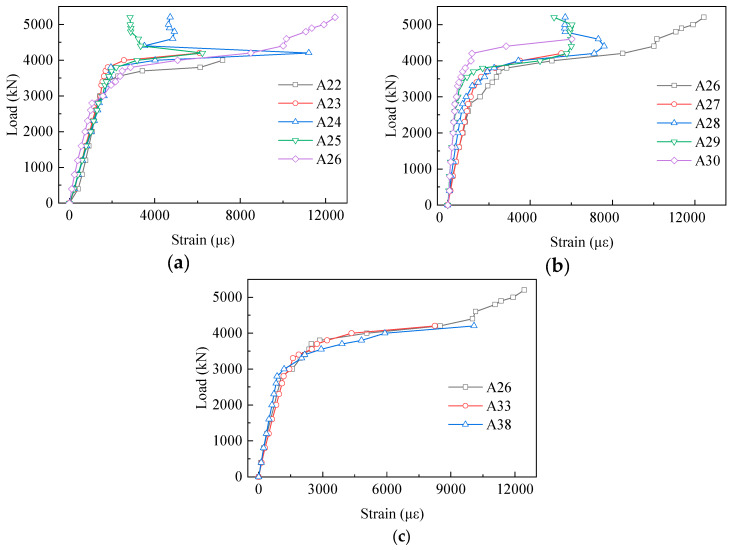
Load–strain curves of the exposed gusset plates: (**a**) A22 to A26 in the fourth row; (**b**) A26 to A30 in the fourth row; and (**c**) A26 to A38 in the middle column.

Figure 10 presents the strain results of the gusset plates that were exposed to the air. Different from the strain results of the embedded gusset plates, the exposed gusset plates had a higher strain level. From the results in Figure 10a,b, the strains increased sharply after 3800 kN. Moreover, almost all the strain results of the fourth-row gauges exceeded the yield strain at the load of 4000 kN. Interestingly, the strains of the embedded gusset plates declined gradually from the middle to the edge in the loading direction. In the vertical direction, Figure 10c shows the load–strain curves of the middle row, where no significant variation tendency from A26 to A38 was evident. However, it should be noted that three measuring points were at a high-strain level. The strain of A26, A33, and A38 were bigger than the yield strain, when the load was about 3500 kN. Furthermore, the strain of those three points reached 8489 µε, 8249 µε and 10085 µε, respectively, at the load of 4200 kN. In addition, the gauges could not function well soon afterward. These results suggest that the exposed part of the gusset plates was a key area for the steel–concrete composite truss joint to exert bearing capacity.

### 3.5. Load–Strain Curves of the Truss-Web Members

**Figure 11:**
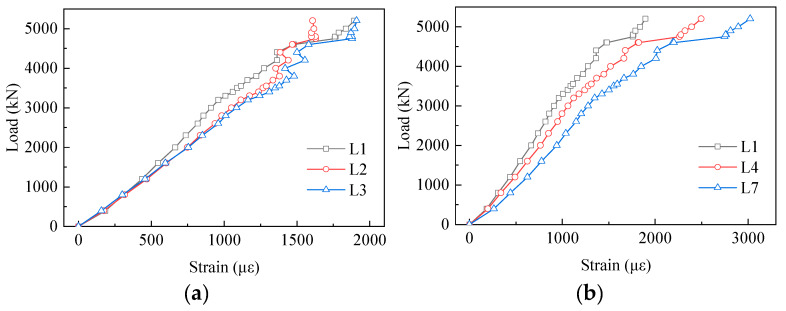
Load–strain curves of the tensile truss-web member: (**a**) L1, L2, L3; and (**b**) L1, L4, L7; L1: No. 1 strain gauge of tensile truss web.

Figure 11 shows the measured strains of the tensile truss-web members. Almost all the strains of the tensile truss-web members were smaller than the yield strain during the whole loading process. From Figure 11a,b, it can be seen that the strain results at each loading stage and the trend of increasing were different. The slope of the load–strain curves decreased gradually, which showed that the strains of the tensile truss web increased along the loading direction. By contrast with L1, the tensile strain of L2 and L3 increased by 16% and 24% at the load of 3500 kN, respectively. In the vertical direction, Figure 11b shows that the strains increased from L1 to L7 and the growth trend was more obvious. Evidently, what stands out in the Figure is the growth of the strains in both directions.

**Figure 12:**
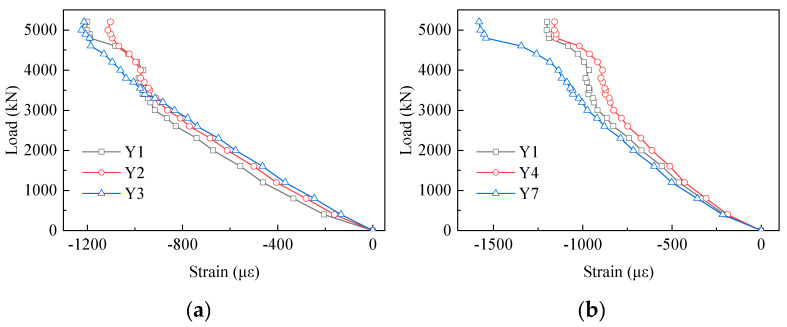
Load–strain curves of the compressive truss-web member: (**a**) Y1, Y2, Y3; and (**b**) Y1, Y4, Y7; Y1: No. 1 strain gauge of compressive truss web.

The strain results of the compressive truss web are displayed in Figure 12. As shown in Figure 12a, the strain value decreased from Y1 to Y3 along the loading direction, which was contrary to the strain distribution of the tensile truss web. The striking observation to emerge from the strain comparison between the tensile truss web and the compressive truss web was that the area of truss web near the inside of the angle had greater strains. Moreover, the regularity of the strain distribution in the vertical direction was weaker in comparison with the tensile truss web.

### 3.6. Load–Strain Curves of PBL Shear Connectors

**Figure 13:**
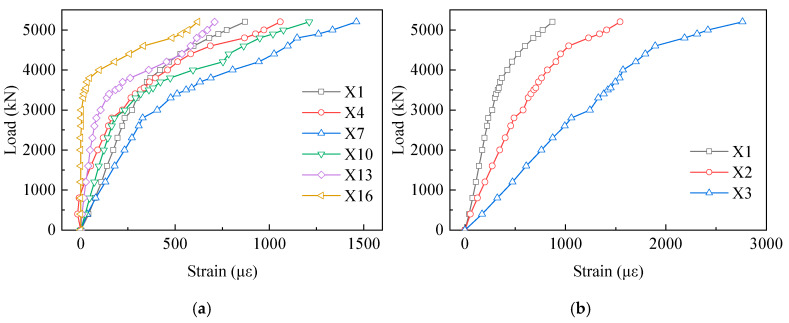
Load–strain curves of the perfobond rib (PBL) shear connectors: (**a**) X1 to X16 in the first row; and (**b**) X1 to X3 in the first column.

Figure 13 displays the strain results of the PBL shear connectors. From this chart, we can see that no measuring points in the first row exceeded the yield strain during the loading process. Furthermore, there was a visible increase in the strains from X1 to X7 in the first row. Compared with X1, the strain of X7 increased by 71% at the load of 3500 kN. However, on the contrary, the strains decreased from X7 to X16. Compared with X7, the strains of X10, X13 and X16 reduced by 35%, 67% and 96% at the load of 3500 kN, respectively. The strains of the measuring points in the first row increased firstly and then decreased along the loading direction. In the vertical direction, in Figure 13b there was a clear trend of increase in the strain from X1 to X3. The strains of X2 and X3 were about 2.11 and 4.36 times that of X1 at the load of 3500 kN, respectively.

**Figure 14:**
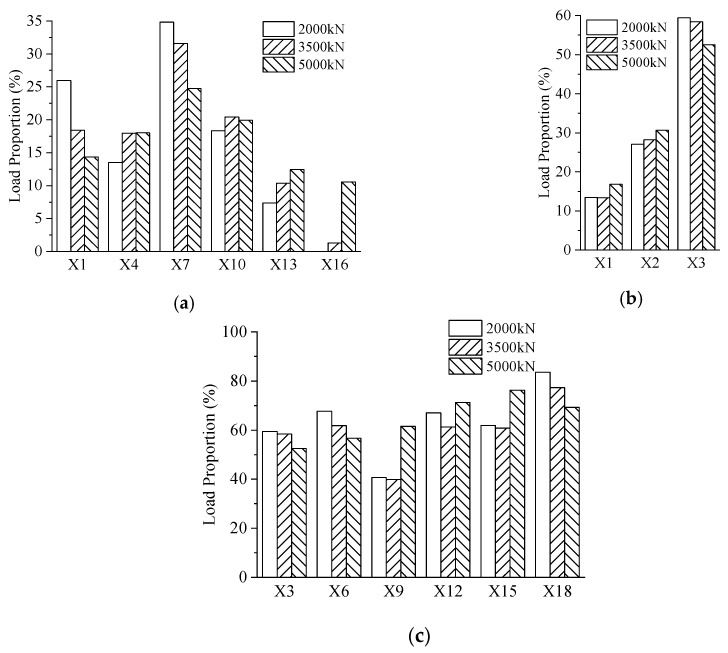
Load proportion of the PBL shear connectors: (**a**) X1 to X16 in the first row; (**b**) the first column; and (**c**) the load proportion of the strain gauges in the third row to their respective columns.

Figure 14 shows the load proportion of PBL shear connectors in the same row and the same column with different loads. In the first row, the load proportions from X1 to X16 were 26%, 14%, 35%, 18%, 7% and 0%, respectively, at the load of 2000 kN. The PBL shear connectors in the first row took on 14%, 18%, 25%, 20%, 12%, and 11% of the strains in the first row, when the load reached 5000 kN. Hence, in the same row, it can be seen that the first three PBL shear connectors in the first row bore the vast majority of the load in the initial stage. Moreover, the load proportion of the first three PBL shear connectors decreased gradually with the increase in the load. As shown in Figure 14b, at the load of 2000 kN, the load proportions of X1, X2, and X3 were 14%, 27%, and 59%, respectively. What stands out in Figure 14b is the rapid increase in the load proportion in the vertical direction. Moreover, the load proportion of X3 exceeded 50%. Figure 14c presents the load proportion of the third row of measure points in its column. The load transmitted by the third of points accounted for at least 60% of the total load transmitted by the same row of PBL shear connectors excluding X9. It was suggested that the third row of PBL shear connectors played the vital role in transferring the load between the concrete chord and the gusset plates.

## 4. Conclusions

In this investigation, the purpose was to fully study the mechanical behavior and failure mode of steel–concrete connection joints by static model tests, especially to determine the strain distribution rule of the main components and the typical failure mode of such joints. The following conclusions can be drawn based on the present study.

1. Compared with the previous experimental results in Table 3, the present results indicated that the proposed joints showed excellent bearing capacity and deformability. The yield load of Specimen 1 and Specimen 2 was 3200 kN and 3400 kN, respectively. The ultimate load of Specimen 1 and Specimen 2 was 5200 kN and 5400 kN, respectively. Furthermore, the ultimate displacement of Specimen 1 was 59.01 mm and the ultimate displacement of Specimen 2 reached 68.31 mm.
2. In the experiments, Specimen 1 and Specimen 2 shared similar failure modes, such as the fracture of gusset plates, the slippage of high-strength bolts, the local buckling of compressive splice plates, the fracture of tensile splice plates and concrete cracking. In particular, the fracture of gusset plates, the slippage of high-strength bolts and concrete cracking were the most prominent failure modes. Moreover, it can be seen from the comparison of the failure modes between the proposed joints and the previous test specimens that the fracture of the gusset plates and the slippage of the high-strength bolts seemed to be two typical failure modes of such connection joints with high-strength bolts.
3. The strain distribution of the steel–concrete connection joint followed certain rules. For the concrete chord, the strains decreased quickly in the loading direction. On the contrary, the strains increased in the vertical direction. For the gusset plates, in the loading direction, the strains reduced gradually from the middle to the edge. In the vertical direction, strains of the embedded gusset plate enhanced from the top to the bottom, but the strain variation tendency of the exposed gusset plate was not obvious. For the steel truss-web members, the areas near the inside of the angle between the tensile truss web and the compressive truss web had greater strain values than in other areas. For PBL shear connectors, the strain distribution patterns were in keeping with the patterns of the embedded gusset plates. The third-row PBL shear connectors resisted most of the load rather than the other two-row connectors.
4. The exposed gusset plate was the key component of the steel–concrete connection joint to resist the load. The exposed gusset plate was at a higher strain level than the other members at the same loading step. Under the extreme condition, the exposed gusset plates of both specimens experienced a severe tensile fracture and were therefore unable to continue to bear the load. Furthermore, in the ultimate state, the fracture of the gusset plates prevented the specimen from continuing to bear the external load.

In this study, we focused on the experimental investigation of steel–concrete connection joints. However, the numerical analysis is also important for a full understanding of mechanical behavior and failure mode of such joints. We will perform the numerical analysis in another article in detail.

## Figures and Tables

**Table 1:** Material properties of the test specimen.

Member	Material	$f_{y}$ (MPa)	$f_{u}$ or $f_{cu}$ (MPa)	$E_{s}$ or $E_{c}$ (GPa)
Concrete	C50	N/A	61.3	34.6
Gusset plate	Q370qE	452	583	206
Steel web	Q370qE	439	557	206
Reinforcement	HRB400	458	640	203

$f_{y}$: yield strength of steel; $f_{u}$: tensile strength of steel; $f_{cu}$: compressive strength of concrete; $E_{s}$: elastic modulus of steel; $E_{c}$: elastic modulus of concrete. N/A: this item does not exist.

**Table 2:** Comparison of the failure modes.

Joint Type	Specimen	Concrete Chord (mm)	Gusset Plate (mm)	Truss Web (mm)	Failure Modes
A	RGP-1 [23,30]	517 × 833	12	217 × 183 × 12	5,7
	RGP-2,3 [23,30]	517 × 833	12	217 × 183 × 22	1,2
	SJ-1 [18]	367 × 400	8	217 × 183 × 12	8
	SJ-2 [18]	367 × 400	8	217 × 183 × 12	1,2
	Specimen 1	334 × 367	16	268 × 184 × 20	1,2,3,4,6
	Specimen 2	334 × 367	16	268 × 184 × 20	1,2,3,4,6
B	JSGP-1 [23]	517 × 833	N/A	217 × 183 × 12	5
	JSGP-2 [23]	517 × 833	N/A	217 × 183 × 22	6
	PSGP-1 [23]	517 × 833	N/A	217 × 183 × 12	5
	PSGP-2,3 [23]	517 × 833	N/A	217 × 183 × 22	6
	ZHJD-1,2 [31]	367 × 400	N/A	217 × 183 × 12	5,6
	EHT [11]	2200 × 250	N/A	Φ318 × 15	6
	T1 [32]	225 × 325	N/A	Φ180 × 8	6

Joint type A: the gusset plates and the truss-web members are connected by high-strength bolts. Joint type B: the truss-web members are partially embedded in the concrete chord, and there is no gusset plate. Please refer to the corresponding reference for the detailed definition of RGP, SJ, JSGP, PSGP, ZHJD, EHT, and T1. SJ-2: Strengthened the gusset plates. 1: Fracture of gusset plates. 2: Slippage of high-strength bolts. 3: Local buckling of compressive splice plates. 4: Fracture of tensile splice plates. 5: Local bucking of compressive web member. 6: Concrete cracking. 7: Yield of tensile web member. 8: Local buckling of gusset plates.

**Table 3:** Experimental characteristics of the steel–concrete composite joint specimens.

Specimen	*N* *_y_*	*N* *_u_*	*N* *_u_/N* *_y_*	*D* *_y_*	*D* *_u_*	*D* *_u_/D* *_y_*	*EI*
RGP-1 [23]	3200	3500	1.09	27.03	-	-	11.84
RGP-2 [23]	3400	4200	1.24	23.50	42.79	1.82	14.47
RGP-3 [23]	3400	4500	1.32	23.43	43.46	1.85	14.51
SJ-1 [18]	2156	2548	1.18	16.13	34.49	2.14	13.37
SJ-2 [18]	2156	2940	1.36	15.92	40.12	2.52	13.54
Specimen 1	3200	5200	1.63	10.90	59.01	5.41	29.36
Specimen 2	3400	5400	1.59	12.89	68.31	5.30	26.38

*N_y_* and *D_y_*: yield load (kN) and the corresponding displacement (mm); *N_u_* and *D_u_*: ultimate load and the corresponding displacement; *EI* (the joint stiffness): *N_y_ /D_y_* (10^4^ kN /m).
